# Vaginal Dysbiosis from an Evolutionary Perspective

**DOI:** 10.1038/srep26817

**Published:** 2016-05-26

**Authors:** Natalia Schlabritz-Loutsevitch, Scott E Gygax, Edward Dick, William L. Smith, Cathy Snider, Gene Hubbard, Gary Ventolini

**Affiliations:** 1 Texas Tech University Health Sciences Center at the Permian Basin, Odessa, Texas, USA; 2 Femeris Women’s Health Research Center, Genesis Biotechnology Group - Hamilton, New Jersey, USA; 3 Southwest National Primate Research Center, San Antonio, Texas, USA; 4 University of Texas Health Sciences Center, San Antonio, Texas, USA.

## Abstract

Evolutionary approaches are powerful tools for understanding human disorders. The composition of vaginal microbiome is important for reproductive success and has not yet been characterized in the contexts of social structure and vaginal pathology in non-human primates (NHPs). We investigated vaginal size, vulvovaginal pathology and the presence of the main human subtypes of *Lactobacillus* spp./ BV-related species in the vaginal microflora of baboons (*Papio spp*.). We performed morphometric measurements of external and internal genitalia (group I, n = 47), analyzed pathology records of animals from 1999–2015 (group II, n = 64 from a total of 12,776), and evaluated vaginal swabs using polymerase chain reaction (PCR) (group III, n = 14). A total of 68 lesions were identified in 64 baboons. *Lactobacillus iners, Gardnerella vaginalis, Atopobium vaginae, Megasphaera I*, and *Megasphaera II* were not detected. *L. jensenii, L. crispatus*, and *L. gasseri* were detected in 2/14 (14.2%), 1/14 (7.1%), and 1/14 (7.1%) samples, respectively. *BVAB2* was detected in 5/14 (35.7%) samples. The differences in the vaginal milieu between NHP and humans might be the factor associated with human-specific pattern of placental development and should be taken in consideration in NHP models of human pharmacology and microbiology.

Microbial involvement is essential for the reproductive success of the host^1^. The composition of the human vaginal microbiome is critical for maintaining the first line of defense against pathogens^2^. The landscape of the vaginal microbiome depends on socio-economic conditions, country of origin, promiscuity, hormonal status, and other factors^3^. An abnormal microbiome composition is associated with such pathological conditions as bacterial vaginosis, vulvar pain^4^, susceptibility to sexually transmitted diseases (STD) and non-sexually transmitted diseases, infertility and adverse pregnancy outcomes^5^.

Evolutionary approaches are powerful tools for understanding human disorders. Baboons (*Papio spp.*, an Old World non-human primate (NHP)) are extensively evaluated and used in reproductive research^6^,^7^. A key difference between the vaginal microbiomes of human and NHPs is the universal dominance of lactobacilli in humans, in contrast to the relative paucity of these species in NHPs^8^,^9^. However, the subtypes of the vaginal microbiome have not yet been characterized in the contexts of social family structure and vaginal pathology in *Papio* spp. This information is essential to understanding the pathophysiology of human disorders and to develop effective treatment strategies. Although one of the important factors influencing microbial diversity is vaginal size^8^,^10^, there have been no reports on this parameter in baboons. In the present study, we aimed to investigate vaginal size, vulvovaginal pathology and the presence of the main human subtypes of *Lactobacillus* spp.–*L. crispatus, L. gasseri, L. jensenii and L. iners*^11^–in the vaginal microflora of baboons.

## Results

### Morphometry of baboon external genitalia

The mean diameter of the introitus was 1.33 ± 0.6 cm, the mean distance from the cervix to the introitus was 6.88 ± 1.7 cm, and the mean distance from the introitus to the fornix was 7.45 ± 1.7 cm. The mean ano-genital distance was 2.38 ± 1.2 cm (all data are presented as the mean ± SEM).

### Pathology of the vagina and vulva

A total of 68 lesions were observed in 64 baboons (from total n = 12,776, where “n” is the total number of morphologic diagnoses in baboons at Texas Biomedical Research institute from 1999 through 2015.) (Table 1). The most common pathological findings were vaginal stenosis (n = 19), vulvar ulcers (n = 19) and inflammatory changes (vaginitis (n = 11) and vulvitis (n = 6)). Vaginal stenosis, vulvar ulcers, vulvitis, vaginal ulcers, and vulvar strictures were presumed to be sequelae of *Herpesvirus papio* 2 (HPV2) infection^12^,^13^,^14^,^15^ and combined represented 69% (n = 47) of total lesions observed. Only one case of vaginitis was cultured and yielded beta-hemolytic *Streptococcus* spp. Four neoplasms were identified: two papillomas and one myxoma in the vagina and a squamous cell carcinoma involving the vulva.

#### Lactobacillus and Bacteroides species

The age, reproductive history, housing, and PCR findings for the baboons from which vaginal swabs were collected and evaluated by PCR analysis for lactobacilli and pathological bacterial subspecies are summarized in Table 2. *L. iners, Gardnerella vaginalis, Atopobium vaginae, Megasphaera I* and *Megasphaera II* were not detected in the specimens studied. *L. jensenii, L. crispatus*, and *L. gasseri* were detected in 2/14 (14.2%), 1/14 (7.1%), and 1/14 (7.1%) samples, respectively. *BVAB2* was detected in 5/14 (35.7%) samples. Four BVAB2-positive animals were housed in the same harem cage. The tuf PCR was negative for other *Lactobacilli spp.*

## Discussion

Host-microbiome interactions are critical for host development. The reproductive evolution of the host is accompanied by microbial evolution and vice versa^16^. Numerous examples of this microbial evolution have recently been reported for baboons and include Papio-unique *Brucella* sub-species^17^,^18^ and papilloma and HPV2^19^. The definition of “normal” vaginal microbial communities differs among species. A healthy human vaginal environment is characterized by the dominance of lactobacilli^20^,^21^. These lactobacilli transform glycogen into lactic acid, generating an acidic environment^22^ and forming protective biofilms^23^ that prevent the colonization and proliferation of potentially pathogenic organisms.

NHPs may rely on different defense mechanisms for protection against sexually transmitted diseases. The differences between humans and NHPs include the vaginal pH (acidic in humans (pH < 4.5)^22^ and acidic-alkaline in baboons (pH = 5.5–6.5^24^), the anatomy of the utero-cervical junction (sharp anterflexio in women compared to “scarcely noticeable” ventroflexio in baboons)^25^, and increased diversity of microbial communities in baboons compared to humans^24^. Interestingly, microbial diversity in primates is determined by the size of the vagina (or baculum length)^8^. The length of the vagina is 10–12 cm in humans^26^ and approximately 7 cm in baboons in our study. The discrepancies between published observations (decreased microbial diversity despite increased vaginal size in humans) could be explained, among others things, by the great ability for the vagina to stretch^25^ and increase vaginal size due to sexual swelling^27^ in baboons. Additionally, social structure and copulative behavior of baboons and humans also differ^28^. Baboons live in harem communities (one male and typically 10–15 females), and males require several vaginal introductions before ejaculation. In general, the specific social structure and higher promiscuity might have been important for promoting species development^29^,^30^. A comparison of the general distribution of parasites between NHP and humans revealed a relative abundance of fungi and bacteria (22% and 38%, respectively) in humans compared to NHPs (3% and 10%, respectively)^31^. These differences in the overall microbial landscape may be responsible for the development of specific local, including vaginal, protective mechanisms. Interestingly, the differences in vaginal lactobacilli between baboons and humans are not accompanied by differences in vaginal fungal composition^32^.

The histological and cytological changes of the vagina during the menstrual cycle are similar in humans and baboons^33^, including an increased level of glycogen-enriched cells during ovulation^33^,^34^. Differences in the structural morphology of the vagina include epithelial maturation (which occurs in the early proliferative phase in baboons but the ovulation phase in humans), the absence of erythrocytes in the vaginal smear around ovulation^35^ and the presence of cornification of the vaginal epithelium in 10% of baboon specimens^36^; in humans, hyperkeratosis represents a metaplastic change^37^. In *Papio* spp. the microbial milieu does not change upon the administration of exogenous progestins and is independent of menstrual cycle phases^9^,^24^, whereas levonorgestrel therapy and menstrual cycle phases are associated with changes in microbial communities in humans^38^,^39^. Evolutionary pressure may have resulted in the formation of hormone-sensitive microbial communities.

The frequencies of vaginal and vulvar pathologies among all pathological diagnoses in baboons are 0.6% and 0.04% (respectively)^40^. In our study, the most common pathology was vaginal stricture (45%), presumably associated with HPV2 (Simian agent 8)^12^. The disease, which is the most common STD in captive baboons, has devastating consequences in *Papio* spp., preventing intercoitus^14^. However, recent publications have suggested that these lesions may also be associated with *Treponema* infection^41^,^42^. The course of infection with herpesvirus simplex is not as devastating in humans^43^, possibly due to the protective role of *L. crispatus* during viral infection. Conversely, the clinical course of infection with *Treponema pallidum* in baboons^41^,^44^,^45^ is mild compared to that in humans. Baboons have not been reported to have STDs caused by Ureaplasma, *Gardnerella vaginalis, Atopobium vaginae*, or *Megasphaera I*. In agreement with this observation, we did not detect these four species in our sample set. Interestingly, in contrast to humans, baboons do not exhibit increased numbers of infection-related stillbirths and preterm births^25^,^46^.

**Figure 1:**
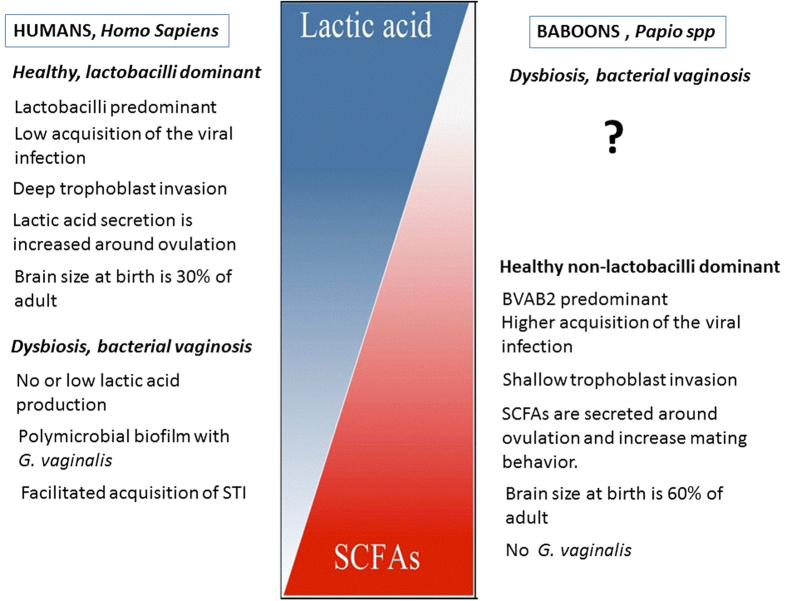
Comparison of the vaginal environment and selected reproductive differences between baboons (*Papio spp.*) and humans. Blue represents an acidic and red represents an alkaline environment (modified from^63^).

The abundance of lactobacilli in our study (21.5%) is in agreement with a previous report^9^ in which lactobacilli were detected in 16% of wild-caught baboons but lower than the rate reported by Skangalis *et al.* (47.4%)^47^. *L. crispatus* is one of the most frequently detected phylotypes in the human vaginal microbiome (85%)^11^, but is among the lactobacilli with the lowest abundances in baboons^8^. In agreement with this observation, *L. crispatus* was detected in only one animal in our study (7.1%), a young female in a harem cage of 11 females. Yildirim *et al.* detected *L. crispatus* in olive but not yellow baboons^8^. The species in our study are hybrids of yellow, olive, and hamadryas baboons; therefore, it is difficult to draw conclusions regarding the specificity of subspecies. In Rhesus macaques (another Old World NHP), the abundance of *L. crispatus* is much lower (0.65%)^48^, and *L. johnsonii*^49^ and *L. reuteri*^48^ are predominant. In humans, *L. crispatus* protects against *G. vaginalis*^50^, which has not been detected in the baboon vaginal microbiome. Remarkably, the genome of *G. vaginalis* includes the tetracycline resistance gene (tet(M)). This gene is also detected in *N. gonorrhoeae* and *U. urealyticum*, vaginal microbial species that are present in humans but absent in baboons^51^. However, the tetM gene was the most abundant gene in vaginal swabs of wild and captive baboons^52^. The source of this gene remains to be elucidated. *L. crispatus* protects against viral infection^50^,^53^. Viral infection of cytotrophoblasts decrease their invasive capacity^54^, leading to shallow trophoblast invasion. Trophoblast invasion in baboons is shallow in contrast to deep invasion in humans^55^. In humans, the abundance of *L. crispatus* may decrease the viral load and thus promote trophoblast invasion (Fig. 1).

According to a phylogenetic tree, *L. iners and L. gasseri* are related species^56^; however, *L. iners* was not detected in the samples in our study, whereas *L. gasseri* was present in 2/14 samples. In macaques, *L. iners* was not detected, but *L. gasseri* was present in 2/304 samples, and the most common was *L. johnsonii* (85/304^48^), which is related to *L*. *iners* and *L. gasseri. L. iners* has the shortest genome^57^ and is dominant in Caucasian/Asian women (34.1%)^58^, whereas *L. gasseri* is present at a much lower abundance (6.3%)^58^. Considering the evolution of macaques, baboons and hominids^59^,^60^, the absence of *L. iners* might be the result of intra-species evolution.

In humans, bacterial vaginosis is associated with an abundance of *Megasphaera* type I, BVAB2, *Gardnerella vaginalis and Atopobium vaginae*^61^. *Megasphaera* type I, BVAB2, and *G. vaginalis* are rare or absent in sexually unexposed women. In our study, we did not detect *G*. *vaginalis*, *Atopobium vaginae*, and *Megasphaera type* I in baboons. In agreement with observations in humans, the majority of BVAB2-positive animals (four out of five) were multiparous 14- to 15-year-old animals, an age comparable to perimenopause in humans^62^. Only one nulliparous young animal was BVAB2-positive, which was attributed to the housing of this baboon in the harem cage with the other BVAB2-positive animals. The diagnosis of BV is non-existent in NHPs. Interestingly, the majority of the vaginal anaerobic flora in baboons is represented by the common species of BV in humans (*Sneathia* from the phylum Fusobacteria^24^). These microbes produce short chain fatty acids (SCFAs)^63^, volatile substances, which stimulate the mating behavior of NHPs^64^. Lactobacilli and an acidic environment in the vagina may be predisposing factors for the acquisition of BV in baboons.

In conclusion: our study confirmed the low abundance of human-specific *Lactobacillus* spp. in baboons. The absence of *L. iners, Gardnerella vaginalis, Atopobium vaginae*, and *Megasphaera I* in the vaginal microflora of *Papio* spp. is a novel finding. The presence of lactobacilli might indicate a predisposition to BV in NHPs.

## Materials and Methods

### Animal characteristics, housing and handling

#### Overall study design

This study included three groups of baboon, hybrids of yellow, olive, and hamadryas baboons (*Papio spp*.). In group I, morphometric measurements of external and internal external genitalia were obtained during bi-annual health checks (n = 16) or necropsy (n = 31). In group II, animals with available pathology records on pathological vulvar and vaginal changes were retrospectively analyzed (n = 64). In group III, vaginal swabs from baboons obtained during health exams were analyzed by polymerase chain reaction (PCR) (n = 14).

#### Group composition and animal housing

**Group I.** Baboons were housed in two open-top 6-acre metal and concrete corrals with dirt floors and gang cages with concrete floors at the SNPRC (Southwest National Primate Research Center, Texas Biomedical Research Institute) as previously described)^65^ **Group II.** Pathology records of animals housed at SNPRC from 1999–2015 were retrospectively analyzed. **Group III.** Vaginal swabs of 14 baboons (*Papio spp*.) housed in harem cages at the SNPRC were collected during routine reproductive examinations (n = 12) or necropsy (n = 2). All animal care procedures were approved by the Animal Care and Use Committee of the Texas Biomedical Research Institute, which is accredited by the International Association for the Assessment and Accreditation of Laboratory Animal Care, in accordance with the approved guidelines.

### Morphometry of external genitalia

**Figure 2:**
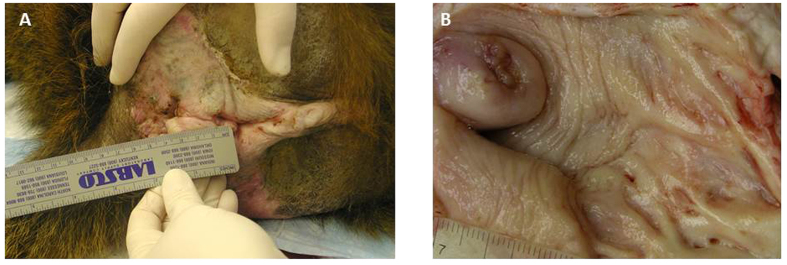
External (**A**) and internal (**B**) measurements, performed in baboons (*Papio spp*).

Animals were sedated via intramuscular injection of ketamine (10 mg/kg) as described previously^65^. The ano-genital distance was measured with a measuring tape from the middle of the anus to the middle of the introitus. The diameter of the introitus was measured from the upper to the lower pole (Fig. 2A). During necropsy, the length of the vagina was measured using a ruler from the introitus to the cervix (introitus to cervix distance) and to the left fornix (introitus to fornix distance) (Fig.2B).

### Collection of vaginal specimens

Vaginal specimens were collected using sterile cotton swabs after the perineal skin was cleaned with Betadine solution and rinsed several times with sterile saline solution. Specimens were stored at −80 **°**C until further evaluation (8–9 years).

### Polymerase chain reaction

A real-time PCR (qPCR) assay was used to detect and determine the relative concentrations of the vaginal flora as described previously^66^,^67^. The qPCR assays identified vaginal *Lactobacillus* spp., including *L. crispatus*, *L. gasseri*, *L. iners*, and *L. jensenii*. The assays also detected facultative anaerobic bacteria (*Gardnerella vaginalis*, *Atopobium vaginae (*AV), bacterial vaginosis-associated bacteria (BVAB2), and *Megasphaera* I and II). qPCR analysis of gene transcripts was performed using a Bio-Rad iCycler RealTime PCR machine and 2× Taqman Master Mix. RNA was extracted using TRIzol (Invitrogen, Carlsbad, CA). Primer probe sets were designed in-house using the software packages Primer ExpressTM v2.0 (Applied Biosystems) and Beacon Designer v2.0 (PREMIER Biosoft International). Additionally PCR, detecting tuf gene, encoding elongation factor Tu, from 33 strains representing 17 Lactobacillus gene target was performed^68^.

## Additional Information

**How to cite this article**: Schlabritz-Loutsevitch, N. *et al.* Vaginal Dysbiosis from an Evolutionary Perspective. *Sci. Rep.* **6**, 26817; doi: 10.1038/srep26817 (2016).

## Figures and Tables

**Table 1:** Lesions of the vulva and vagina in the baboon colony housed at the Southwest National Primate Research Center (1999–2015).

Organ	No. cases	% of cases
**Vagina**		
Constriction/Stenosis/Stricture	19	45.24
Vaginitis	11	26.19
Ulcer	4	9.52
Hyperplasia	2	4.76
Papilloma	2	4.76
Prolapse	2	4.76
Adenosis	1	2.38
Myxoma	1	2.38
**Total**	**42**	**100**
**Vulva**		
Ulcer	17	65.38
Vulvitis	6	23.08
*Histoplasma duboisii*	1	3.85
Squamous cell carcinoma	1	3.85
Stricture	1	3.85
**Total**	**26**	**100**

Note: With the exception of a single biopsy (vaginal papilloma), all diagnoses were made at necropsy. Four animals had two diagnoses each at necropsy: two baboons had vaginitis and vulvitis, one animal had a vaginal ulcer and a vulvar ulcer, and one had vaginitis and vaginal stenosis.

**Table 2:** Age, reproductive history, housing, and PCR findings for baboons with vaginal swabs.

	Age (years)	Number of pregnancies/stillbirths	Number of females in the group at the time of analysis^1^	Pregnant (yes/no)	Lactobacillus spp.***	Human BV associated species****
1	6	1/0	11*	yes	none	none
2	16	4/0	11*	no	*L. crispatus*	none
3	12	6/0	11*	yes	none	none
4	12	7/0	11*	yes	none	none
5	9	5/0	11*	no	none	none
6	15	2/0	11*	no	*L. jensenii*,*L. gasseri*	*BVAB2*
7	5	0/0	16**	no	none	*BVAB2*
8	12	3/0	16**	no	none	none
9	15	6/0	16**	n/a	none	*BVAB2*
10	16	6/2	16**	yes	*L. gasseri*	none
11	14	2/0	16**	no	none	*BVAB2*
12	11	5/0	16**	no	none	none
13	17	6/0	N/A	Yes, early abortion	none	none
14	14	4/0	N/A	yes	none	*BVAB2*

^1^The animal population was split between two harem cages, animals marked * were housed in one cage, animals marked ** were housed in another cage.

^***^L. crispatus, L. gasseri, L. jensenii, L. iners.

^****^BV– bacterial vaginosis associated bacteria. *Gardnerella vaginalis, Atopobium vaginae, Megasphaera type I, Megasphaera type II*, and *Bacterial Vaginosis Associated Bacterium 2 (BVAB2)*.
